# An empirical investigation of determinants & sustainability of public debt in Pakistan

**DOI:** 10.1371/journal.pone.0275266

**Published:** 2022-09-28

**Authors:** Sundus, Samina Naveed, Tanweer Ul Islam

**Affiliations:** Department of Economics, National University of Sciences and Technology, Islamabad, Pakistan; University of Almeria, SPAIN

## Abstract

An assessment of debt dynamics and its sustainability is very important in formalizing prudent and effective macroeconomic policies especially for the economies with weak macroeconomic fundamentals and alarming debt levels. Keeping in view the recent debt escalation in Pakistan, this study aims to explore the important factors that influence the public debt dynamics in case of Pakistan and to evaluate its sustainability. This study applies the debt dynamic approach for empirical assessment of drivers of changing debt levels and analysis of public debt sustainability. Furthermore, ARDL approach is utilized to study the short- and long-run debt dynamics using historic data from 1975 to 2021. This study is distinct from already existing work on debt assessment in Pakistan as it examines both important dimensions of public debt (determinants & stability) by employing the novel dynamic debt modelling approach and using most recent data. The study finds a positive and significant impact of fiscal deficit, exchange rate depreciation and interest rate on public debt in Pakistan. The debt sustainability analysis also reveals the instability of public debt for the entire study period except for few years. The regression results corroborate with the findings from stability analysis, and the main driving forces for increasing the debt burden of the country are found to be the fiscal indiscipline along with the rising cost on account of ER depreciation and higher interest rates.

## 1. Introduction

An evaluation of determinants of debt dynamics and debt sustainability is vital for designing prudent macroeconomic policy for any economy. Its importance for finance constrained and heavily indebted countries become more significant, where root cause of majority of the macroeconomic imperfections is highly linked to escalating debt levels. Debt sustainability thus become a primary requisite for macroeconomic stability in those economies as capital accumulation and economic growth are hampered on account of high levels of public debts [1–3]. Developing or poor countries are normally a victim of this menace, as financially strong economies can rationally mange to service their debt obligations. Therefore, an assessment of debt dynamics and debt sustainability for economies that face persistent budget deficits along with weak macroeconomic fundamentals and rising debt levels would be very pertinent in formalizing prudent and effective macroeconomic policies for those economies.

Pakistan is one of those countries where persistent budget deficits have led to high levels of indebtedness and debt to GDP ratio has increased from almost 60 percent in 2010 to 72 percent in 2021 [4]. This rising level of indebtedness has not only led to macroeconomic imperfections but can brings the country under the threat of insolvency. Hence, it become pertinent under the given scenario that an assessment of debt substantiality in case of Pakistan is conducted. However, before the assessment of debt sustainability, it would be quite insightful if we are able to determine the core drivers or factors that affect the debt dynamics of a country. The debt dynamics are extensively investigated in the literature and plethora of studies have been conducted to determine the core factors affecting the rising level of debt. The important factors responsible for rising debt levels are found to be the primary balance, interest rate, exchange rate, economic growth, the output gap and inflation [5–8].

Besides the literature on debt dynamics, there are number of studies that have conducted the debt sustainability analysis [6, 9–12]. The vast literature on debt dynamics in the context of Pakistan is also not an exception and there re no. of studies which have extensively covered the assessment on rising level of debts. However, those studies are either limited to the determinants of debt dynamics [13–17] or a separate assessment of debt sustainability [17–22]. Besides, mostly the literature focus on external debt and its sustainability with the scant literature on dynamics of total public debt and its sustainability analysis [16, 21, 23]. However, for a financially starved country like Pakistan facing twin deficits, the high level of public indebtness is a serious macroeconomic issue. This chronic and persistent problem of high level of indebtness for Pakistan thus necessitates to conduct research which should not only determine the core factors responsive for rising level of public debt but simultaneously assess the sustainability of the debt in the current scenario. Therefore, the objective of current research is to determine the important factors or determinants that influence the public debt dynamics in case of Pakistan and to evaluate the debt sustainability during 1975–2021.

This study contributes to literature in several dimensions. First, it covers both important dimensions of assessment of public debt- determinants & stability, for Pakistan. Earlier literature investigates the determinants of debt [13–17] or a separate evaluation of sustainability [17–22]. Second, we utilize the novel dynamic debt modelling proposed by Mupanga & Roux [6] in context of Pakistan. Furthermore, this study exploits the latest updated historic data to explore the major drivers of rising debt burden in case of Pakistan.

Rest of the study is organized as follows. Section 2 covers the review of existing literature on debt dynamics and debt sustainability analysis. Section 3 includes the analysis regarding the composition of public debt in Pakistan. Section 4 incorporates empirical methodology for determining debt dynamics as well as debt stability along with the data description. While Section 5 discusses the regression results along with an analysis of stability results. Section 6 finally close the entire discussion with the conclusion of the study.

## 2. Literature review

The empirical literature on debt is extensive and covers many dimensions ranging from an impact of accumulation of debt on economic growth of the countries to an investigation of determinants of debt burden and an analysis of debt sustainability. The economic growth can be either accelerated or hampered on account of many factors and there is extensive literature on factors affecting economic growth of the nations [24–28]. However, in particular context to debt, the economic growth is hindered on account of high level of indebtness [1–3]. The driving factors for changing debt levels have been examined widely in the literature and the studies on debt sustainability is also extensively available. Piscetek [5] determines the primary balance as an important contributor to changing debt levels, while the effect of other determinants like interest-growth differential and exchange rate is found be benign. While fiscal pressures result in escalating debt levels for the countries when examined at macro levels, however, studies also validate the positive impact of fiscal pressures in improving the financial performance of the companies at micro level analysis [29]. Besides, the fiscal pressure also creates a very strong short term and long-term impact on the financial equilibrium of the publicly traded companies [30]. Debt dynamics are also influenced by output gap as well as huge stock flow adjustments that are needed to finance social and political expenditures [6]. A panel study by Campos *et al*. [7] using a sample of 117 countries found the debt dynamics to be explained by change in exchange rate, interest rate, inflation, and growth rate of the economy. The debt dynamics are also significantly influenced by interest rate growth differential in case of most African countries [8].

Besides the literature on debt dynamics, there are number of studies that have conducted the debt sustainability analysis [9, 10, 12, 31, 32]. These studies mostly employ the fiscal reaction function and conducts a stochastic analysis of debt. The assessment of debt is also thoroughly covered for Pakistan but the existing studies are confined either to an investigation of determinants of debt accumulation or to an analysis of debt sustainability. The existing literature identifies different core factors responsible for either the accumulation of debt or a decline in debt burden. Bilquees [13] and Chandia and Javed [17] found the primary deficit and ER changes as the main determinants of debt accumulation in Pakistan. These findings are further supported by Awan *et al*. [16], however, their study find an additional impact of trade openness on debt burden of Pakistan. Contrary to these findings are the results by Akram [14] where a positive impact of economic growth and stability is determined in reducing the debt burden. The literature on debt sustainability employing the fiscal reaction function has mostly found the debt levels to be unsustainable or weakly sustainable in case of Pakistan [17–21]. These studies do not cover the forecasting and prediction of sustainability of public debt, however, Ejaz and Haider [22] examine the projection of debt for 2019–2025 and find Pakistan’s debt to be unsustainable for this forecast period.

Review of literature points to this important fact that a thorough investigation of core determinant of rising public debt along with the debt sustainability analysis have not been conducted in a single study for Pakistan. Hence, it is imperative to examine both these dimensions by using latest data for Pakistan as escalating debt levels have become a critical problem for Pakistan economy.

## 3. Analysis of composition of public debt in Pakistan

**Fig 1:**
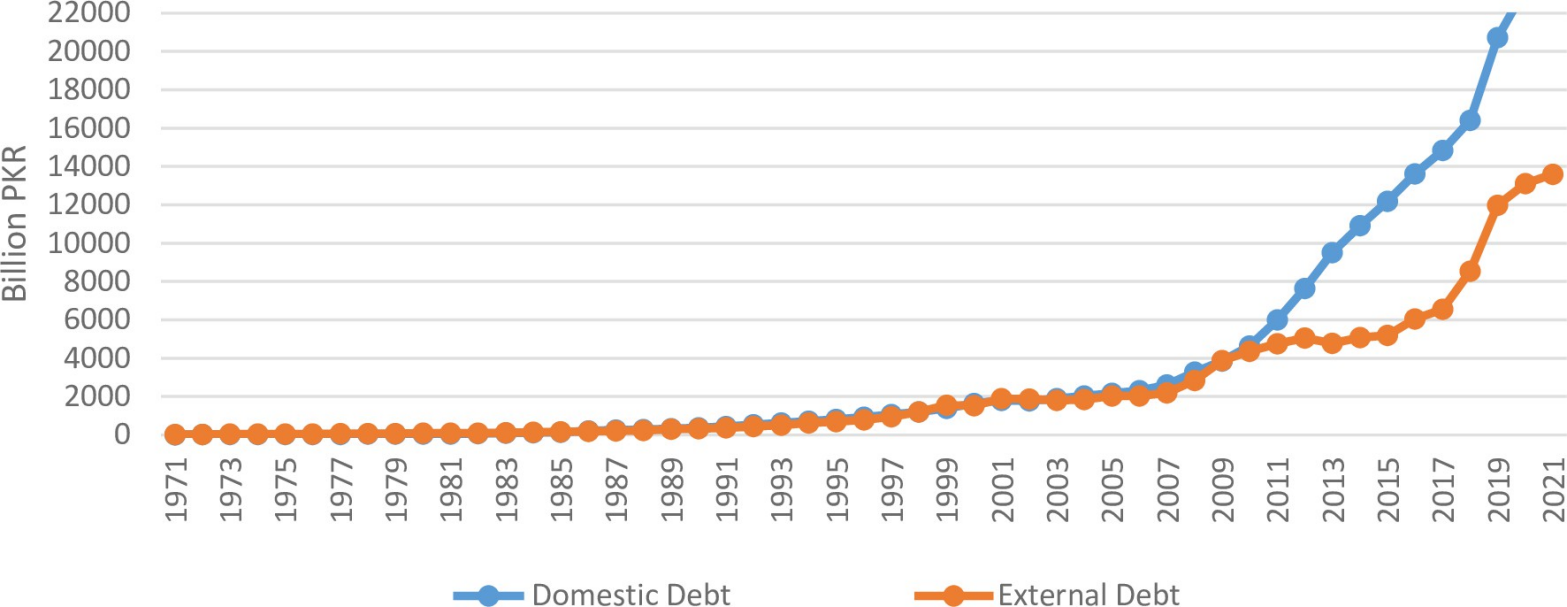
Debt trend.

**Fig 2:**
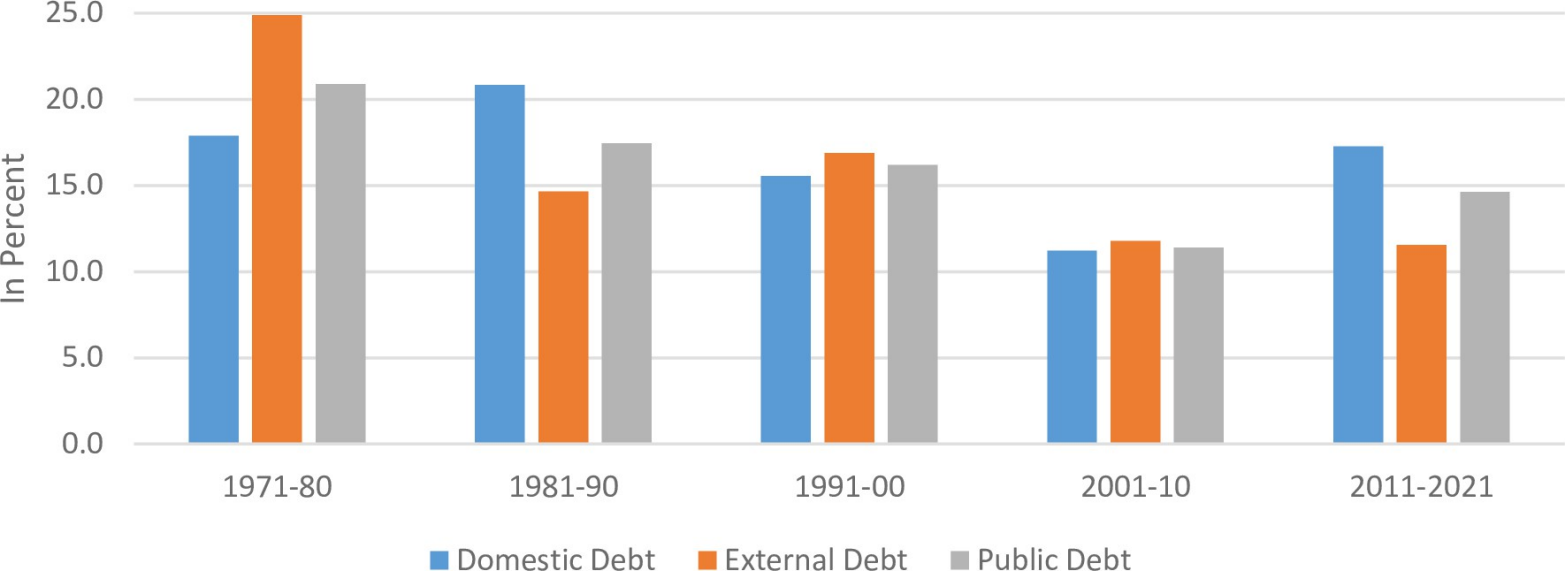
Decade wise debt growth rates.

This section attempts to explain the trend and composition of domestic and external debt of Pakistan. Before the last decade, Pakistan was doing well in terms of debt sustainability [33], however, debt (both domestic & external) escalated sharply in the last decade (Fig 1). The growth rate of domestic debt during the last decade is as high as 17.3 percent while external debt growth is 11.6 percent (Fig 2). This raises serious questions regarding the sustainability of debt for Pakistan’s economy with average GDP growth rate of 3.72 percent in the same period.

Currently, Pakistan is heavily relying on domestic debt which is almost two-third of gross public debt [4]. State Bank of Pakistan is pursuing tight monetary policy to curb the ever-increasing inflation leading to high debt servicing burden at domestic level. Exchange rate depreciation, on the other hand, exhausting the debt servicing capacity of Pakistan.

**Fig 3:**
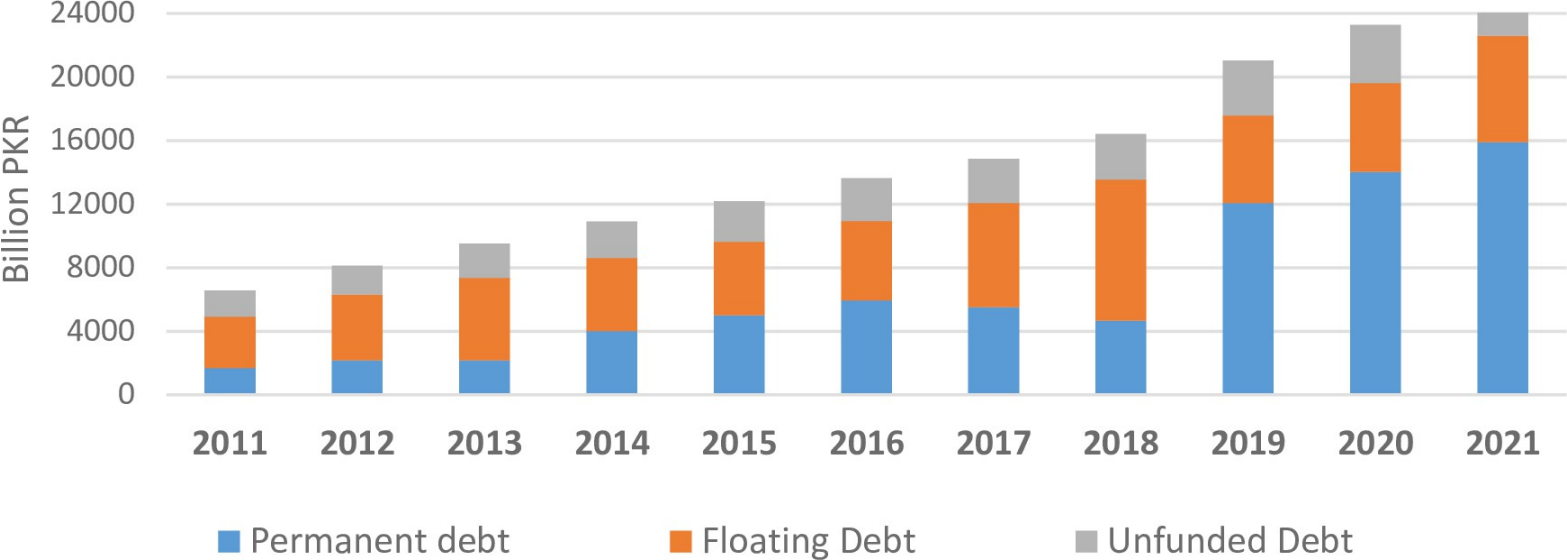
Domestic debt composition.

Fig 3 categorizes the domestic debt in terms of permanent, floating, and unfunded debt. The permanent debt is the government’s long-term debt consisting of medium to long term instruments (Pakistan Investment Bonds (PIBs), Government Ijara Sukuk (GIS), Prize Bond etc.). Floating debt consists of short-term debt such as Market Treasury Bills (MTBs) and State Bank borrowing through purchase of Market Related Treasury Bills (MRTBs). While the unfunded debt is a Medium-term debt which comprises of voluntary savings schemes like the National Savings Scheme instruments. It is well evident from Fig 3 that floating debt constitute the largest part of domestic debt portfolio during 2011–2018, followed by permanent debt. However, from 2019–2021, this composition has changed and permanent debt has become the major constitute of total domestic debt followed by floating debt. The unfunded debt constitutes only a meagre share of total domestic debt during the entire period of 2011–2021.

**Fig 4:**
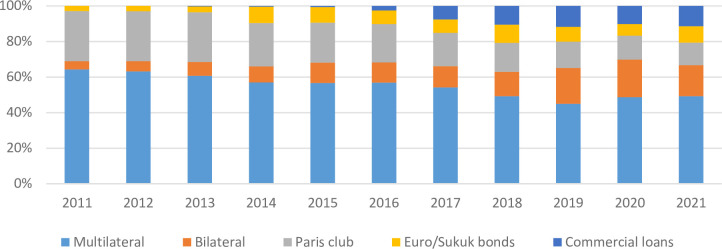
External debt composition.

Fig 4 summarizes external debt which is mostly accounted for by multilateral lenders (the World Bank, International Monetary Fund, Asian Development Bank), bilateral lenders (Paris Club), and commercial lenders. The largest contribution to external debt is attributed to the multilateral lending sources followed by commercial loans. Since external debt needs to repay in foreign currency, so deprecation of domestic currency (Pakistani rupee) has strong bearing in raising the external debt cost.

A brief analysis of the public debt in terms of its composition clearly attribute the rising debt burden to domestic borrowing whose debt service obligation is further deteriorated by rising interest rates. Besides, the significant domestic currency depreciation further adds to external debt burden, consequently escalating the public debt.

## 4. Empirical methodology and data description

### 4.1. Empirical model

This study primarily employs the public debt dynamics model developed by Mupunga and Raux [6], where change in the public debt is attributed to key macroeconomic variables such as primary balance, real GDP, exchange rate and real interest rate. Along with these core variables identified by Mupunga and Raux [6], the other important determinants affecting debt dynamics of a country are also utilized in empirical testing. These potential determinants or control variables are output gap and non-interest current account balance. The following empirical model incorporates the core determinants affecting the debt dynamic in case of Pakistan.


$${\Delta D}_{t}=\beta_{0}+\beta_{1}{PB}_{t}+\beta_{2}R_{t}+\beta_{3}G_{t}+\beta_{4}{ER}_{t}+\beta_{5}\mu_{t}+{\beta_{6}X}_{t}+\epsilon_{t}$$ (1)

In Eq (1), the subscript ‘*t*’ represents time, Δ*D* is the change in the total public debt as a percentage of GDP, PB is the primary balance as a percentage of GDP, R is the real effective interest rate, G is the real GDP, ER is the exchange rate, μ is the seigniorage, and X is a vector of control variables which consists of output gap (Gap) and non-interest current account balance as percentage of GDP (NICAB) and ϵ_t_ is the white noise process.

To examine the impact of explanatory variables on change in debt to GDP ratio in Eq (1), the study employs ARDL bounds testing approach. This approach is not only valid for mix order of integration, but it also examines both the short run and long run relationship between the variables under study. Thus Eq (1) can be modified into the following unrestricted error correction model (ECM) to test the long-run relationship among the variables of interest.


$${\Delta D}_{t}=\beta_{0}+\sum_{i=1}^{p}{\beta_{1i}\Delta D}_{t-i}+\sum_{i=1}^{p}{\beta_{2i}\Delta PB}_{t-i}+\sum_{i=1}^{p}{\beta_{3i}\Delta \mu }_{t-i}+\sum_{i=1}^{p}\beta_{4i}\Delta R_{t-i}+\sum_{i=1}^{p}{\beta_{5i}\Delta G}_{t-i}+\sum_{i=1}^{p}{\beta_{6i}\Delta ER}_{t-i}+\sum_{i=1}^{p}\beta_{7i}{\Delta NICAB}_{t-i}+\sum_{i=1}^{p}{\beta_{8i}\Delta Gap}_{t-i}+\lambda_{1}{\Delta d}_{t-1}+\lambda_{2}{PB}_{t-1}+\lambda_{3}\mu_{t-1}+\lambda_{4}R_{t-1}+\lambda_{5}G_{t-1}+\lambda_{6}ER_{t-1}+\lambda_{7}{NICAB}_{t-1}+\lambda_{8}{Gap}_{t-1}+u_{t}$$ (2)

Where all *β*’s express short run dynamics of the model, all *λ*’s represents the long run relationship and *p* represents the optimal lag length which will be based on AIC criteria. The rejection of the following null hypothesis based on *F-test* implies the existence of long-run relationship.


$$H_{0}\;:\;\lambda_{1}=\lambda_{2}=\lambda_{3}=\cdots =\lambda_{8}=0$$

In the presence of long-run relationship, the error correction form of Eq (2) can be written as follows:

$${\Delta D}_{t}=\sum_{i=1}^{p}{\alpha_{1i}\Delta D}_{t-i}+\sum_{i=1}^{p}{\alpha_{2i}\Delta PB}_{t-i}+\sum_{i=1}^{p}{\alpha_{3i}\Delta \mu }_{t-i}+\sum_{i=1}^{p}\alpha_{4i}\Delta R_{t-i}+\sum_{i=1}^{p}{\alpha_{5i}\Delta G}_{t-i}+\sum_{i=1}^{p}{\alpha_{6i}\Delta ER}_{t-i}+\sum_{i=1}^{p}\alpha_{7i}{\Delta NICAB}_{t-i}+\sum_{i=1}^{p}{\alpha_{8i}\Delta Gap}_{t-i}+\eta {ECT}_{t-1}+\mu_{t}$$ (3)

Where, *ECT*_*t*−1_ is the disequilibrium term and *η* is the speed of adjustment towards equilibrium. It indicates how much of the disequilibrium is being corrected, that is, the extent to which any disequilibrium in the previous period is being adjusted in current point.

### 4.2. Debt sustainability analysis

Eq 1 examines the core determinants that affect the debt dynamics of the country; however, it does not incorporate the debt sustainability assessment. The assessment of sustainability requires further investigation in terms of examining whether the debt is stable or not. To determine the stability, following budget constraint can be written in the presence of external financing and considering the currency depreciation [6].

$${Debt}_{t}=(1-\alpha )(1+i_{t}^{d}){Debt}_{t-1}+\alpha (1+i_{t}^{f})(1+\varepsilon_{t}){Debt}_{t-1}-({pb}_{t}+{\Delta m}_{t})+{sf}_{t}$$ (4)

where, *α* is the share of foreign debt and (1-*α*) is the share of domestic debt. The superscripts, *d* & *f* represent the domestic and foreign variables. To get the stock of public debt to GDP, divide Eq (4) by nominal $\mathrm{GDP}=P_{t}Y_{t}=(1+\pi_{t})(1+g_{t})P_{t-1}Y_{t-1}$

$$D_{t}=\frac{\left(1-\alpha )(1+i_{t}^{d}\right)}{\left(1+\pi_{t})(1+g_{t}\right)}D_{t-1}+\frac{\alpha (1+i_{t}^{f})(1+\varepsilon_{t})}{\left(1+\pi_{t})(1+g_{t}\right)}D_{t-1}-({PB}_{t}+\mu_{t})+{SF}_{t}$$ (5)

where, *π*_*t*_ is inflation rate, *g*_*t*_ is growth rate of real GDP, and upper-case letters represent variables in terms of nominal GDP ratios. Using simple algebra, we may rewrite the above equation as follows

$$D_{t}=\varphi_{t}D_{t-1}-({PB}_{t}+\mu_{t})+{SF}_{t}$$ (6)

where, φt=[(1+itw)+α(1+itf)εt](1+gt)(1+πt) is automatic debt dynamics and $i_{t}^{w}=[(1-\alpha )i_{t}^{d}+\alpha i_{t}^{f}]+\alpha \varepsilon_{t}(1+i_{t}^{f})$ is the effective nominal interest rate which is the weighted sum of domestic and foreign interest rate and depends on exchange rate. Following Allen (2008), $i_{t}^{f}$ is allowed to be approximated by $i_{t}^{w}$ denoted by ${\hat{r}}_{t}$ yields

$$\varphi_{t}=\frac{\left[{\hat{r}}_{t}-\pi_{t}(1+g_{t})-g_{t}+\alpha \varepsilon_{t}(1+{\hat{r}}_{t})\right]}{\left(1+g_{t}+\pi_{t}+g_{t}\pi_{t}\right)}$$

The stability of debt depends upon automatic debt dynamics, *φ*_*t*_, r_*t*_, and g_*t*_. For the stable debt level, *φ*_*t*_<1, implying that public debt to GDP ratio ultimately falls to a predetermined threshold level. While for the explosive case, *φ*_*t*_>1 means the ratio of public debt to GDP would explode from the predetermined threshold level. This indicate that if the real interest rate which the government pays on its debt is greater than the growth rate of GDP, the interest burden on existing debt increases, whereas the debt to GDP ratio also increases. At the same time the debt to GDP ratio grows without bound, which is clearly unstable, if a government borrows for servicing of existing debt. Therefore, primary balance surplus requires to at least cover the interest payments due for the stability of public debt.

### 4.3. Data description

The analysis is based on an annual data set for Pakistan over 47 years from 1975 to 2021. It has been collected from various sources due to unavailability of data for all variables from a single source. The description of variables along with their data sources are provided in Table 1 below.

**Table 1:** Description of variables.

Variable	Data source	Description
Public debt to GDP, *d*_*t*_	Pakistan Economic Survey	It is measured as change in total public debt to gross domestic product
Primary Balance to GDP *PB*_*t*_	State Bank of Pakistan	Primary balance to GDP can be calculated as ratio of total revenue minus noninterest expenditure to GDP.
Output Gap Gap_t_	Constructed by HP filter	It is measured as deviation of estimated output from potential output
Seignorage μ_*t*_	State Bank of Pakistan	It is measure of change in monetary base as percentage of GDP
Noninterest current account balance NICAB	State Bank of Pakistan	It is obtained by excluding interest payments on foreign debt from the current account balance.
Real GDP growth *G*_*t*_	World Development Indicators	It is a measure of the rate of change that a nations GDP experiences from one year to another, expressed as percentage.
Real effective interest rate R_t_	Pakistan Economic Survey	It is obtained by dividing the interest payments in the current period by the previous period’s debt stock.
Exchange rate *ER*_*t*_	International Financial Statistics	It is the price of one country’s currency in term of another country’s currency *i*.*e*. national currency per U.S dollar.

## 5. Results & discussion

### 5.1. Stationarity test

In case of time series data, choice of the appropriate econometric technique critically depends upon the stochastic properties of the variables under consideration. This study employs ADF test to check the stationarity of the variables and the results are reported in Table 2. All the variables are found to be stationary at level except primary balance to GDP that is stationary at first difference. The results of unit root test through ADF confirms a mix order of integration for the variables. For mix order of integration data, ARDL is the natural choice to examine the long- and short run relationship between the variables [34].

**Table 2:** ADF unit root test.

Variable	At Level		At First Difference	
	Without trend	With trend	Without trend	With trend
D	-6.969***	-6.883***	--	--
PB	-2.646	-2.614	-9.080***	-9.266***
NICAB	-3.44**	-3.383*	--	--
GAP	-3.696***	-3.601**	--	--
μ	-6.168***	-6.342***	--	--
R	-6.614***	-6.747***	--	--
G	-5.07***	-5.553***	--	--
ER	-6.004***	-5.934***	--	--

***, **, *denote statistical significance at the 1%, 5% and 10% level

The descriptive statistics for all the variables of the model are reported in Table 3. The important observation from these descriptive stats is that none of the series deviate from normal distribution except for interest rate. However, non-normality of any regressor is not a concern as the sum of random variables tends to be normal (Central Limit Theorem). Further, the errors of the model follow the assumption of normality (Table 7). Correlation matrix (Table 4) indicates that some of the regressors have significant association. However, keeping in mind the time series data properties, these correlations are not threatening as far as multicollinearity is concerned. The variance inflation factor (VIF) values for all variables are less than the benchmark value- five which indicates no problem of multicollinearity (Table 4, last column). On balance, these statistics suggest that the estimated parameters would be unbiased, consistent, and efficient.

**Table 3:** Descriptive statistics.

	PB	μ	D	NICAB	GAP	R	ER	G
Mean	-1.90	1.88	73.23	-2.08	0.00	-2.01	2.05	3.04
Median	-1.97	1.77	67.40	-2.00	0.00	-1.88	1.35	3.14
Maximum	1.91	3.85	100.30	6.30	0.02	3.64	8.21	5.91
Minimum	-7.69	0.21	52.10	-8.10	-0.02	-22.13	-1.72	0.17
Std. Dev.	2.35	0.77	13.23	3.19	0.01	3.94	2.33	1.23
Skewness	-0.44	0.39	0.30	0.41	0.24	-2.86	0.86	-0.01
Kurtosis	2.71	2.73	1.70	3.46	2.57	15.87	2.92	2.72
Jarque-Bera	1.67	1.33	3.89	1.70	0.79	379.95	5.63	0.15
Probability	0.43	0.51	0.14	0.43	0.68	0.00	0.06	0.93
Observations	47	47	47	47	47	47	47	47

**Table 4:** Correlation matrix & VIF.

Correlation	ER	G	MU	NICAB	OG	PB	R	VIF
ER	1							1.31
G	0.034	1						2.13
μ	-0.143	0.279	1					1.20
NICAB	-0.314*	0.150	-0.171	1				2.61
OG	0.267	0.364	0.146	-0.508*	1			2.01
PB	0.055	-0.033	-0.419*	0.523*	0.000	1		3.54
R	-0.207	0.095	-0.178	0.058	0.046	0.062	1	3.50

* Indicates the significance at 5 percent level.

### 5.2. Regression results

The econometric testing under ARDL comprises of two-step procedure where the first step involves the identification of long run relationship between the variables of the study, while the second evaluates the long run and short run impact of explanatory variables on the dependent one. The F- statistics (4.92) is greater than the upper bound value at 5 percent level of significance (3.5) implying rejection of the null hypothesis of no cointegration. This confirms the existence of cointegration relationship between the variables of the study and helps us to move to the next step of estimating the long run and short run coefficients of the model. The study estimates both long-run and short run elasticities using Eqs (2) and (3). Tables 5 and 6 present the estimated results of long- and short run models.

**Table 5:** Long run results.

Variable	Coefficient	P-value
PB	-0.95***	0.0007
Gap	4.42	0.9578
R	1.57***	0.0000
G	0.50	0.4303
ER	1.43***	0.0000
NICAB	-0.05	0.8252
μ	- 1.31	0.3149

The dependent variable is change in public debt to GDP. The ***, **, * denote statistical significance at the 1%, 5% and 10% level.

Respectively.

**Table 6:** Short run results.

Variable	Coefficient	P-value
D (PB)	-1.18***	0.0008
D (Gap)	-116.12	0.3218
D (Gap(-1))	-15.59	0.9205
D (R)	1.32***	0.0000
D (R(-1))	-0.30**	0.0282
D (R(-2))	-0.40***	0.0050
D (G)	-0.44	0.5042
D (ER)	0.97***	0.0008
D (μ)	0.16	0.8426
D (μ (-1))	2.20*	0.0026
D (μ (-2))	-1.71**	0.0358
D (NICAB)	0.37	0.2783
D (NICAB(-1))	-0.42	0.3094
ECT-1	-1.24***	0.0000

The ***, **, * denote statistical significance at the 1%, 5% and 10% level.

It is well evident from the long run results (Table 5) that primary balance, real interest rate, and exchange rate has a significant impact on raising the debt levels in case of Pakistan. Primary balance to GDP which is commonly considered as the most important contributor in escalating debt burden in developing countries turns out to be a significant contributor in raising debt burden in case of Pakistan economy. As 1 percent increase in primary deficit leads to increase the debt to GDP by 0.95 percent in long run. This result is supported by the fact that primary balances do have a strong bearing on debt dynamics and Pakistan’s primary deficits and fiscal indiscipline throughout the period under consideration has act as a catalyst in accelerating country’ debt obligations. This result is consistent with the findings of Chandia and Javid [17] and Tahir and Tahir [35].

The result for ER validates the strong impact of ER depreciation on increasing debt burden of the country, as 1 percent depreciation contribute to increasing debt obligation by almost 1.43 percent. It implies that a fall in the value of Pak Rupee over the long run has resulted in increasing the cost of borrowing and value of external debt in terms of local currency, thereby contributing to public debt burden of the country. This result supports the analysis in section 3 (composition of public debt), regarding the role of external debt in escalating public debt. This result is further in line with the findings of Ahmad and Ahmad [18] and Bilquees [13].

The real interest rate also has a positive and significant impact on escalating debt in long run, as 1 percent rise in interest rate leads to increase the debt level by 1.57 percent. This is the variable with the highest magnitude among all the important determinants of debt burden, which implies that higher interest rate leading to an increased cost of borrowing had contributed massively to accumulating more loans to service debt obligations. This finding is reinforced by our earlier discussion in section 3 about the composition of public debt. A brief analysis of the public debt in terms of its composition has clearly attributed the rising debt burden to domestic borrowing whose debt service obligation has further been deteriorated by rising interest rates. Furthermore, this finding is supported by the results of Sheikh *et al*. [36], Babu *et al*. [37] and Chandia and Javid [38]. The impact of economic growth on public debt is found to be contrary to theoretical prediction as an increase in economic growth is associated with an escalation of debt. However, this positive association between these variables is found to be insignificant, implying to reject this causation of relationship. Besides economic growth, the output gap, the noninterest current account balance (NICAB) and seignorage are also found to be the insignificant contributors in rising debt level of Pakistan.

The results of the short run model are presented in Table 6. All the SR results are consistent with the findings of the LR model with the exception of seignorage which was found to be insignificant in LR but comes out as significant determinant in explaining debt dynamics in the SR. Financing budget deficit through seigniorage cause inflation and rise in inflationary pressures compels the Central Bank towards a tight monetary policy stance. This leads to an increase in interest rate, thus escalating the domestic debt servicing cost resulting in an increased debt burden in the short-run. The error correction term is negative and significant, which indicates a reverse correction mechanism and implies a convergence of the system towards stability in case of any disequilibrium.

**Fig 5:**
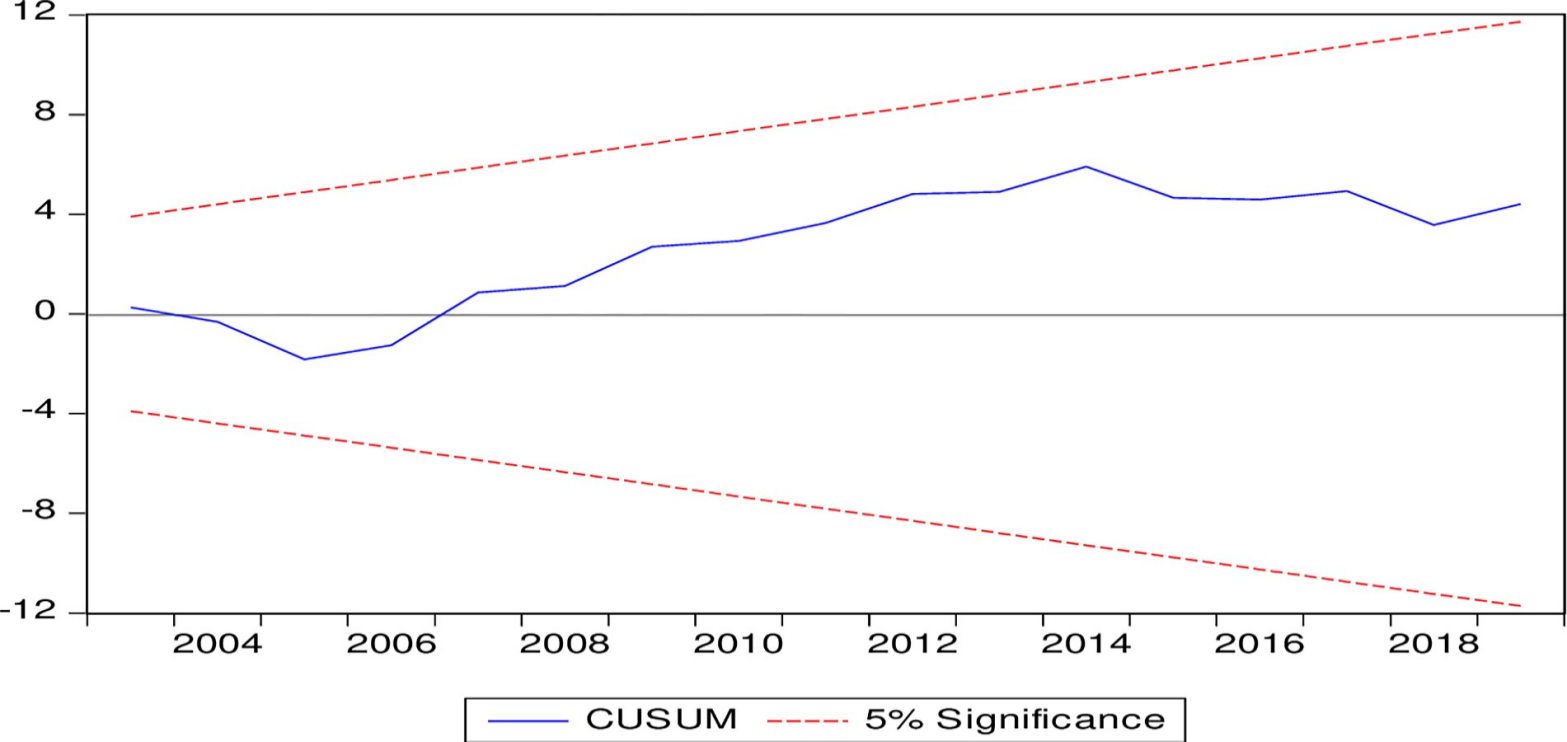
Dynamic stability of the model.

To validates the above findings from our econometric model, diagnostic statistics including CUSUM, Breusch-Godfrey (BP) Serial Correlation test, Breusch-Pagan-Godfrey (BPG) test for Heteroskedasticity, and Hausman’s test of exogeneity are employed (Table 7). The graph of the CUSUM test (Fig 5) shows the stability of the model as the recursive estimates are within the bounds. While both the serial correlation and heteroskedasticity tests are unable to reject the respective null hypothesis of no autocorrelation and homogeneity. This shows that the estimated parameters are efficient. The Hausman’s test establishes the weak exogeneity of the regressor, which implies no problem of endogeneity. Furthermore, the result of JB test for normality confirms that null hypothesis of normality of residuals cannot be rejected, implying that errors follow the assumption of normality (Table 7).

**Table 7:** Autocorrelation, heteroscedasticity & endogeneity test results.

Test	Test Statistic	P-Value
JB Test for Normality	2.04	0.36
Breusch-Godfrey for Serial Correlation	0.95	0.97
Breusch-Pagan-Godfrey for Heteroskedasticity	0.95	0.99
Hausman Test	0.26	0.24

### 5.3. Result of public debt stability

Table 8 reports the results on debt stability for the period of 1982–2021, where the values of Phi less than one indicate debt stability and greater than 1 shows instability of public debt. It is quite evident from the results that out of the total sample of 39 years, the public debt retained a momentum of stability only during a 5-years period (2002–2007). The stability of the debt during this period is attributed to a relatively strong fiscal position of the country along with sustainable external position [39]. Furthermore, Pakistan’s economy started gaining momentum in 2002 onward after a lost decade of 1990s, and this helped the country to record impressive growth performance in subsequent years. This increase in growth was accompanied with an appreciation of the domestic currency which has led to reduce the debt burden of the country and in turn, contributed to the sustainability of public debt. A continuous momentum of debt stability has not been observed for other years covered in the data set, however, some individual years are also the exception during which phi is less than 1 (1987,1990, 2010, 2014, 2016, 2020).

**Table 8:** Public debt stability.

Year	Phi	Year	Phi
1982	8.8349	2002	-1.5718
1983	1.5364	2003	-0.8306
1984	4.0663	2004	0.8976
1985	1.1515	2005	0.2115
1986	2.7127	2006	0.3719
1987	0.3537	2007	0.0748
1988	2.089	2008	6.3548
1989	5.3604	2009	1.3308
1990	0.7655	2010	0.4114
1991	4.1585	2011	1.0385
1992	1.1865	2012	1.8626
1993	6.2872	2013	1.7626
1994	0.7172	2014	-1.0046
1995	3.6642	2015	1.0593
1996	5.7395	2016	-0.0009
1997	3.5503	2017	1.391
1998	1.8056	2018	7.4099
1999	6.722	2019	3.9047
2000	4.476	2020	0.8159
2001	2.2144	2021	2.3138

For rest of the sample years, the Phi is greater than one which implies an instability of the public debt. Among these instable decades, the significant instability is observed for the decade of 1990s. Not only that growth rate of the economy was plummeted to extreme lows during 1990s but there was significant increase in fiscal deficit coupled with external account problem, rapid depletion of the foreign exchange reserves and high inflationary pressures [39]. These factors led the public debt to explode and bring the country to the brink of default. A relatively weak fiscal position and escalating trade deficit has again led to create pressures on currency and has contributed to an explosive nature of debt during the terminal years’ of 2000 decade.

During the period of 2011–2021, for majority of the years, the public debt again shows instability except for three years (2014, 2016 and 2020) and this instability is largely owed to fiscal imbalances along with a rapid depreciation of the currency [4]. An analysis of public debt instability in this section is rationally justified with the results obtained through regression analysis. The rising debt burden in the LR (as reported in section 5.2) was significantly explained by fiscal imbalances, along with the changes in interest rate and exchange rate. The instability in public debt and its explosive nature is also attributed to the fundamental macroeconomic imbalances, along with the fluctuations in exchange rate, as elaborated in this section.

## 6. Conclusion

The escalation of public debt at alarming rates for a finance starved developing country like Pakistan stipulates for an in-depth analysis of determining the core factors responsible for the rise in debt levels. Besides, it is very pertinent to assess the sustainability of current debt to foresee the prudency of macroeconomic policies. In this backdrop, employing the data from 1975–2021, this study has not only attempted to evaluate the important determinants of rising debt levels in Pakistan but also measured the stability of public debt.

The empirical result through ARDL model shows that there is a significant positive impact of fiscal deficit, real interest rate and ER depreciation on rising public debt levels in Pakistan. An increase in primary deficit has a strong bearing on debt dynamics and Pakistan’s primary deficits and fiscal indiscipline throughout the period under consideration has act as a catalyst in accelerating country’ debt obligations. The result for ER validates the strong impact of ER depreciation on increasing debt burden of the country, as fall in the value of Pak Rupee has resulted in increasing the cost of borrowing and value of external debt in terms of local currency, thereby contributing to public debt burden of the country. The real interest rate also has a positive and significant impact on escalating public debt of the country. This is the variable with the highest magnitude among all the important determinants of debt burden, which implies that higher interest rate leading to an increased cost of borrowing has contributed massively to accumulating more loans to service debt obligations.

It is also well evident from the result of stability test that historically, Pakistan’s public debt has been instable and explosive for majority of the years apart from few years. The stable nature of the debt only in few years is attributed to a relatively strong fiscal position of the country along with sustainable external position. However, for majority of the period where an instability in public debt is observed, it is mainly owed to fiscal deficit coupled with external account problems, rapid depletion of foreign exchange reserves and depreciation of currency. These factors have led the public debt to explode and bring the country to the brink of default. These results clearly indicate the need of macro or fiscal adjustments in the economy of Pakistan to reduce the rollover risk and avoiding the high risk of sovereign stress [40].

The results from both the regression and stability analysis corroborate and find out the fiscal indiscipline along with the ER depreciation and interest rates to be the main cause of rising debt burden and instability of public debt. Besides, another important reason for instability is attributed to external account problem. In the light of these results, it is rightly recommended that Pakistan must correct these imbalances and bring fiscal discipline along addressing the external account problems. Pakistan urgently needs wide-ranging structural reforms in the tax system and tax administration to ensure primary surplus to stabilize public debt. Current account deficit should be financed from non-debt creating inflows like FDI and privatization proceeds, grant, and portfolio investment so that the borrowing requirement are minimized and the pressure on domestic currency is also released. Besides, Pakistan also needs to earn enough FX earnings through facilitation of exports, which would assure a strong and stable domestic currency and external account.
