# Correction: An Empirical Investigation of Determinants & Sustainability of Public Debt in Pakistan

**DOI:** 10.1371/journal.pone.0297693

**Published:** 2024-01-19

**Authors:** Sundas, Samina Naveed, Tanweer Ul Islam

The first author’s name is spelled incorrectly. The correct name is: Sundas. The correct citation is: Sundas, Naveed S, Islam TU (2022) An empirical investigation of determinants & sustainability of public debt in Pakistan. PLoS ONE 17(9): e0275266. https://doi.org/10.1371/journal.pone.0275266
